# Embolisation of pulmonary arteriovenous malformations using high-frequency jet ventilation: benefits of minimising respiratory motion

**DOI:** 10.1186/s41747-019-0103-8

**Published:** 2019-07-09

**Authors:** Emanuele Boatta, Roberto Luigi Cazzato, Pierre De Marini, Mathieu Canuet, Julien Garnon, Bob Heger, Thi Mai Bernmann, Nitin Ramamurthy, Christine Jahn, Marc Lopez, Afshin Gangi

**Affiliations:** 1 Service d’Imagerie Interventionelle, Hôpitaux Universitaires de Strasbourg, 1, place de l’Hôpital, 67000 Strasbourg, France; 2 Service de Pneumologie, Hôpitaux Universitaires de Strasbourg, 1, place de l’Hôpital, 67000 Strasbourg, France; 3 Service d’Anesthésie et de Réanimations Chirurgicales, Hôpitaux Universitaires de Strasbourg, 1, place de l’Hôpital, 67000 Strasbourg, France; 4 Department of Radiology, Norfolk and Norwich University Hospital, Colney Lane, Norwich, NR4 7UY UK

**Keywords:** Arteriovenous malformations, Embolisation (therapeutic), Lung, Anesthesiology, High-frequency jet ventilation

## Abstract

**Background:**

To evaluate patient radiation dose and procedural duration recorded during pulmonary arteriovenous malformation (PAVM) embolisation performed using high-frequency jet ventilation (HFJV) as compared with conventional intermittent positive pressure ventilation (IPPV)

**Methods:**

Patients undergoing PAVM embolisation with HFJV assistance after April 2017 were retrospectively identified as group A, and those treated with IPPV before April 2017 as group B. Primary outcomes were patient radiation dose and procedural duration between groups A and B. Secondary outcomes were difference in diaphragmatic excursion between groups A and B, in group A with/without HFJ assistance, technical/clinical success, and complications.

**Results:**

Twelve PAVMs were embolised in 5 patients from group A, and 15 PAVMs in 10 patients from group B. Mean patient radiation was significantly lower in group A than in group B (54,307 ± 33,823 mGy cm^2^ [mean ± standard deviation] *versus* 100,704 ± 43,930 mGy cm^2^; *p* = 0.022). Procedural duration was 33.4 ± 16.1 min in group A *versus* 57.4 ± 14.9 min in group B (*p* = 0.062). Diaphragmatic excursion was significantly lower in group A (1.3 ± 0.4 mm) than in group B (19.7 ± 5.2 mm; *p* < 0.001) and lower with near statistical significance in group A with HFJV than without HFJV (1.3 ± 0.4 mm *versus* 10.9 ± 3.1 mm; *p* = 0.062). Technical and clinical success was 100% in both groups, without relevant complications.

**Conclusion:**

HFJV-assisted PAVM embolisation is a safe, feasible technique resulting in reduced patient radiation doses and procedural time.

## Key points

- Embolisation is the treatment of choice for pulmonary arteriovenous malformations.
- Embolisation of pulmonary arteriovenous malformations can be performed under general anaesthesia and high-frequency jet ventilation.
- This option results in reduced patient radiation dose and procedural time.

## Background

Pulmonary arteriovenous malformations (PAVMs) are anomalous direct communications between pulmonary arteries and veins, comprising single/multiple feeding arteries (classified as “simple” or “complex” types, respectively), an aneurysmal sac/nidus, and one or more draining veins. Eighty per cent of them are associated with hereditary haemorrhagic telangiectasia (HHT), a rare autosomal-dominant disease characterised by recurrent epistaxis, mucocutaneous telangiectasia, and visceral arteriovenous malformations [1, 2]. PAVMs form a right-to-left shunt, potentially resulting in cyanosis, haemorrhage, and significant risk of paradoxical embolism (*e.g.* stroke, cerebral abscess) [3, 4]. Early treatment is usually recommended.

Endovascular embolisation is the treatment of choice and aims to selectively occlude feeding arteries as close as possible to the nidus, while avoiding device migration. Embolisation is typically performed under local anaesthesia and mild sedation. However, despite development of numerous devices facilitating safe, distal vascular occlusion (including microcatheters, detachable coils, microcoils, and vascular plugs [5–13]), procedures may remain challenging due to significant target-lesion motion during respiration. This may preclude two-dimensional (2D)/three-dimensional (3D) roadmap generation, limit precise device navigation/deployment to short periods of controlled apnoea or breathing intervals, and necessitate multiple angiographic acquisitions with increased procedure time and radiation dose. For these reasons, some operators may prefer general anaesthesia with intermittent positive pressure ventilation (IPPV).

High-frequency jet ventilation (HFJV) utilises limited tidal volumes (1–3 mL/kg, often less than dead space) at high frequencies (> 100 cycles/min) to minimise thoracoabdominal motion compared with IPPV [14, 15]. The technique has been applied to thoracic surgery, extracorporeal shockwave lithotripsy, cardiac ablation, and percutaneous tumour ablation procedures to reduce target-lesion motion and optimise therapy [14, 16–25]. To our knowledge, there are no prior studies evaluating potential benefits in endovascular embolisation procedures.

The purpose of this study is to evaluate patient radiation dose and procedural duration recorded during PAVM embolisation performed using HFJV as compared with conventional IPPV.

## Methods

This retrospective study was approved by the institutional review board with a waiver of written informed consent.

### Study population

A retrospective review of a prospectively maintained institutional database was performed to identify consecutive patients undergoing PAVM embolisation under general anaesthesia (mainly due to patients’ refusal of local anaesthesia) between December 2015 and May 2018. Following the introduction of HFJV at our institution in April 2017, all cases undergoing PAVM embolisation under general anaesthesia were systematically treated using HFJV assistance (group A); prior to this, patients undergoing PAVM embolisation under general anaesthesia were treated using conventional IPPV without HFJV (group B).

All patients were affected by HHT and referred for treatment following multidisciplinary discussion between pulmonologists, interventional radiologists, and anaesthesiologists, to prevent future cerebrovascular accident or ameliorate respiratory symptoms. All PAVMs were confirmed on echocardiography/bubble test, demonstrating right-to-left shunts, and contrast-enhanced computed tomography (CECT). No patients in group A had contraindications to HFJV (chronic airways disease with forced expiratory volume in the first second < 1.5, severe obesity, or recent pneumothorax/thoracic surgery).

### Procedures

All procedures were performed on an in-patient basis by the same interventional radiologist (> 7 years of experience in embolisation) under sterile surgical conditions. Procedures were performed in an angiography suite equipped with a flat-panel C-arm cone-beam computed tomography (CBCT) system and XperCT/Embo-Guide tools (Philips Healthcare, Best, the Netherlands). Anticoagulant/anti-platelet therapy and blood clotting parameters were managed according to Society of Interventional Radiology guidelines [26]. Antibiotic prophylaxis (cefazolin, 1 g) and 5000 IU heparin were administered intravenously prior to and during the procedure, respectively.

#### Anaesthesia and HFJV assistance

Following induction of anaesthesia, muscle relaxation, and orotracheal intubation, conventional IPPV was commenced in all patients (6–8 mL/kg predicted body weight tidal volume; respiratory rate adjusted according to end-tidal carbon dioxide [ETCO_2_] level; target 30–40 mmHg). For group A patients, IPPV was discontinued and a HFJV dual-lumen cannula (length 40 cm, diameter 2 mm; Acutronic Medical Systems AG, Hirzel, Switzerland) was introduced into the endotracheal tube. HFJV was initiated using a Monsoon II jet ventilator (Acutronic Medical Systems AG, Hirzel, Switzerland) with frequency 150–250 breaths/min, driving pressure 1–2 bar, inspiratory time 30%, and fraction of inspired O_2_ (FiO_2_) adjusted to maintain peripheral oxygen saturation (SpO_2_) > 95%. Blood pressure, ECG, and SpO_2_ were continuously monitored. ETCO_2_ monitoring was performed intermittently after 5 min and then every 30 min using the jet ventilator capnography device, by injecting five long insufflations with IPPV followed by measurement over 10 s (with the jet switched off). Once embolisation was completed, the HFJV cannula was removed and conventional ventilation was resumed until patient’s awakening/extubation.

#### PAVM embolisation

**Fig. 1:**
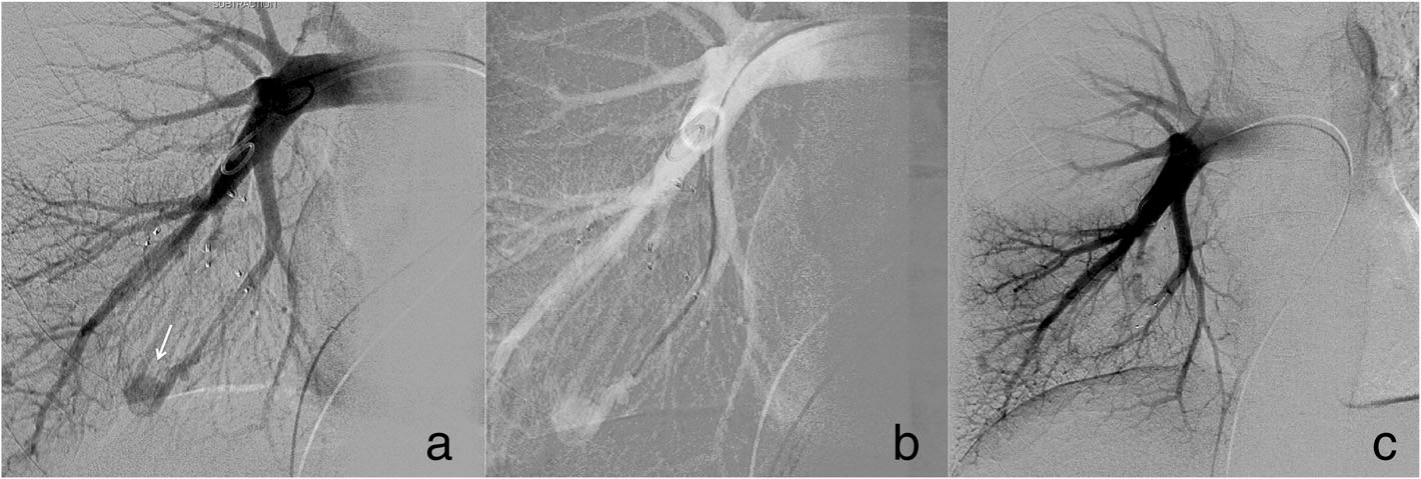
Fluoroscopic images from a group A patient undergoing HFJV-assisted embolisation. **a** Simple PAVM (white arrow) of the right lower lobe identified on initial angiogram. **b** 2D roadmap-assisted selective catheterisation of the feeding artery was performed. **c** Final angiographic check following embolisation with an 8-mm Amplatzer vascular plug IV demonstrates complete vascular occlusion. *HFJV* High-frequency jet ventilation, *PAVM* Pulmonary arteriovenous malformation

Following ultrasound-guided placement of a 6–8-Fr sheath (Pinnacle, Terumo Corporation, Tokyo, Japan) in the femoral vein, a 4-Fr Imager TM II pig-tail catheter (Boston Scientific, Marlborough, MA, USA) was advanced into the target main pulmonary artery, and diagnostic angiography was performed to localise PAVMs and identify number/size of feeding arteries. Feeding arteries were selectively catheterised using a 6-Fr catheter (Neuron catheter, Penumbra Inc., Alameda, CA, USA), and when required, a co-axially 4-Fr MPA 125-cm catheter (Cordis, Johnson & Johnson Company, USA) was used. The 6-Fr catheter was continuously flushed with heparin-saline solution (2500 IU/L) via a Y connector to avoid thrombus formation and minimise neuro-embolic risk. For group A, navigation was assisted by a simple 2D roadmap (Fig. 1) in four patients, and a continuous 3D roadmap automatically delineating feeding arteries (generated by Embo-Guide software following initial 3D rotational angiography with 40 mL of contrast agent at 8 mL/s) was used in one patient. No 2D/3D roadmaps could be generated for group B. Embolisation was performed using conventional or microvascular plugs (MVP, Medtronic, Minneapolis, MN, USA) and/or platinum detachable microcoils with/without hydrogel coating (Ruby Coil, Penumbra Inc., Alameda, CA, USA; Azur Coils, Terumo Corporation, Tokyo, Japan), depending on feeding artery anatomy and available technologies during the study period.

Devices were deployed as distally as possible within feeding arteries, via 4–6-Fr catheters for conventional vascular plugs, or via a co-axial 2.8-Fr microcatheter (Progreat, Terumo Corporation, Tokyo, Japan; inner diameter 0.027″) for microvascular plugs/microcoils. Vascular occlusion was confirmed via selective feeding artery angiogram. A final angiographic check was performed after 3–4 min to confirm satisfactory device position and occlusion. For group B, angiograms throughout the procedure were variably performed with induction of patient’s apnoea, depending on operator requirements. For group A, all angiograms were performed with HFJV, other than a final acquisition under conventional IPPV to compare differences in diaphragmatic excursion.

Following the procedure, patients were admitted to a recovery ward for 24-h observation and discharged when medically fit.

### Follow-up

All patients were clinically reviewed by the treating interventional radiologist and underwent CECT to confirm PAVM occlusion 1 month post-procedure. Serial echocardiography/bubble tests were performed at 1, 6, and 12 months to monitor for right-to-left shunts.

### Data collection and statistical analysis

Patients’ demographics; number, location, and type (simple or complex) of PAVMs treated per session; and type of embolic device were tabulated. Primary outcomes were patient radiation dose and procedural time. Secondary outcomes included mean intraprocedural diaphragmatic excursion, considered as a surrogate for whole-lung movement in group A *versus* B; mean diaphragmatic excursion in group A on final angiogram with HFJV *versus* IPPV; technical success; complications (according to the classification System of the Cardiovascular and Interventional Radiological Society of Europe [27]); and clinical success.

The patient radiation dose was calculated as dose-area product by the proprietary angiographic software (Philips Healthcare, Best, the Netherlands). Procedural time was calculated between the first and last angiographic acquisition.

**Fig. 2:**
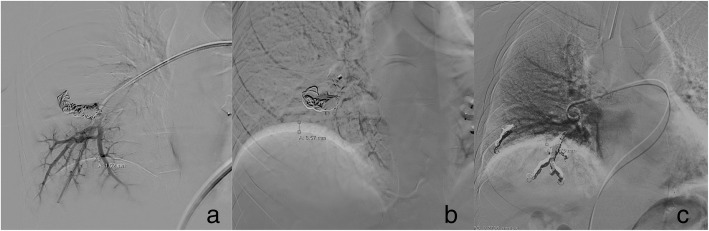
Measurements of diaphragmatic excursion within and between groups. **a** Diaphragmatic excursion (1.52 mm) measured in the cranio-caudal plane during the final angiographic check in a patient from group A under HFJV. **b** A larger measurement (8.57 mm) was obtained in the same patient during the same interventional session with IPPV. **c** Diaphragmatic excursion (11.76 mm) measured in a patient from group B under conventional IPPV. *HFJV* High-frequency jet ventilation, *IPPV* Intermittent positive pressure ventilation

Diaphragmatic excursion was calculated for each case on digital subtracted angiographic series (duration at least 15 s), measuring the cranio-caudal distance between the upper- and lower-most positions of the superimposed hemidiaphragmatic cupolas and recording the lowest measurement for each procedure (Fig. 2).

Technical success was defined as distal occlusion of the feeding artery < 1 cm from the PAVM aneurysmal sac, with complete devascularisation on final angiogram. Clinical success was defined as the absence of neurologic/respiratory symptoms and echocardiographic right-to-left shunt at the last available clinical/sonographic follow-up.

Statistical analysis was performed using R software (R v3.4.5, R Foundation for Statistical Computing, Vienna, Austria). Non-parametric Wilcoxon test was used to compare numerical and discrete variables; *p* values < 0.05 were considered statistically significant.

## Results

In the study period, 20 patients underwent PAVM embolisation. Among them, 15 were treated under general anaesthesia, thus representing the study population. In particular, between April 2017 and May 2018, 5 consecutive patients underwent PAVM embolisation using HFJV assistance (group A). Prior to this, 10 patients were treated using conventional IPPV without HFJV (group B). Mean number of PAVMs embolised per session was 2.4 ± 2.1 (range 1–6) in group A *versus* 1.5 ± 0.7 (range 1–3) in group B (*p* = 0.422). Patient and PAVM characteristics are summarised in Table 1.

**Table 1:** Patient demographics, symptoms, and PAVMs treated in groups A and B

Group	Number of patients	Demographics	Symptoms	Number of PAVMs treated	PAVM location	Embolisation devices
A (HFJV)	5	Age 47.2 ± 11.7 years (34–57 years) 2 males, 3 females	4/5 (80%) 1 prior TIA 3 dyspnoea	12 simple	RLL (*n* = 4) RML (*n* = 1) RUL (*n* = 1) LLL (*n* = 4) LUL (*n* = 2)	6.5-mm MVP (*n* = 4), vascular plug (*n* = 5), coils (*n* = 3)
B (IPPV)	10	Age 41.5 ± 12.4 years (27–65 years) 5 male, 5 female	7/10 (70%) 1 prior TIA 6 dyspnoea	15 (14 simple, 1 complex)	RLL (*n* = 3) RML (*n* = 5) RUL (*n* = 2) LLL (*n* = 3) LUL (*n* = 2)	6.5-mm MVP (*n* = 4), vascular plug (*n* = 3), coils (*n* = 8)

Ages are given as mean ± standard deviation (range). *HFJV* High-frequency jet ventilation, *IPPV* Intermittent positive pressure ventilation, *LLL/LUL* Left lower/upper lobes, *MVP* Microvascular plug, *RLL/RML/RUL* Right lower/middle/ upper lobes, *TIA* Transient ischemic attack

Mean patient radiation exposure was significantly lower in group A than in group B: dose-area product 54,307 ± 33,823 mGy cm^2^ (mean ± standard deviation; range 6135–100,678 mGy cm^2^) *versus* 100,704 ± 43,930 mGy cm^2^ (range 53,109–176,634 mGy cm^2^), respectively (*p* = 0.022).

Procedural time was lower in group A than in group B with a trend towards statistical significance, 33.4 ± 16.1 min (range 16–55 min) *versus* 57.4 ± 14.9 min (range 41–85 min) (*p* = 0.062).

Mean diaphragmatic excursion was significantly lower in group A than in group B (1.3 ± 0.4 mm (range 0.9–1.9 mm) *versus* 19.7 ± 5.2 mm (range 8.7–27.1 mm); *p* < 0.001) and demonstrated a lower trend on final angiograms in group A with HFJV assistance than with IPPV (1.3 ± 0.4 mm (range 0.9–1.9 mm) *versus* 10.9 ± 3.1 mm (range 7.2–15 mm); *p* = 0.062).

Technical success was 100% in both groups without radiologic complications. One patient from group A experienced bronchospasm necessitating temporary switching from HFJV to conventional IPPV, but no episodes of desaturation, hypercapnia, or barotrauma (grade 1 complication) were recorded. All PAVMs were completely occluded on 1-month CECT follow-up without device migration. Clinical success was 100% in both groups on early follow-up (group A: mean 3.75 months, range 1–12 months; group B: mean 16.5 months, range 14–29 months).

## Discussion

The HFJV is a mechanical ventilation method initially described in 1977 by Klain and Smith [28], in which small tidal volumes of pressurised gas are delivered through a narrow-bore endotracheal catheter at very high respiratory rates. The technique facilitates adequate gas exchange while minimising diaphragmatic excursion, and thoracoabdominal target lesions normally subject to significant respiratory positional variation are rendered relatively motionless [14, 15, 25]. Several recent interventional radiology reports have demonstrated improved lesion targeting, reduced technical difficulty and procedural time, and reduced patient radiation dose during thermal ablation of lung, liver, and renal tumours [19–24]. However, to our knowledge, the technique has not yet been reported for endovascular embolisation procedures.

In the present study, HFJV was applied to five cases undergoing PAVM embolisation. Patient radiation dose and procedural time were improved using HFJV compared with IPPV. The presence of nearly static PAVMs enabled generation of accurate 2D/3D roadmaps, facilitating easier catheter navigation and more precise, confident deployment of embolic material. Fewer angiographic acquisitions were required for lesion targeting, significantly reducing radiation dose by 54% and procedural time by mean 24 min (minus 42%, non-significant likely due to small sample size). Similar advantages have been described in prior thermal ablation studies [22–24].

These benefits are likely to be the result of the nearly static PAVMs, and for this reason, diaphragmatic excursion was compared with a historic series of 10 patients undergoing the same procedure using IPPV. As expected, HFJV-assisted cases demonstrated significantly lower diaphragmatic excursion compared to IPPV (mean 1.3 mm, compared with 20 mm for IPPV), and similar results (1.3 mm excursion with HFJV assistance compared with 11 mm for IPPV) were observed intraprocedurally in the five cases with HFJV assistance, although did not reach statistical significance likely due to small sample size. These measurements are comparable with reported excursion of 1–3 cm during quiet breathing and conventional IPPV [29, 30] and with prior studies reporting reduced diaphragmatic motion during HFJV-assisted extracorporeal shockwave lithotripsy [25].

Although direct, reliable/reproducible measurement of PAVM motion was not possible due to retrospective differences in projections/positioning between cases, diaphragmatic excursion was considered an adequate surrogate, since it was less prominently affected by interprocedural factors, and appears to be similar in magnitude to whole-lung movement during respiration [31]. The present measurement of 1.3 mm is comparable to prior reports of HFJV-assisted thermal ablation and radiotherapy procedures. Denys et al. [21] measured target tumour displacement during lung, liver, and renal tumour ablation using CT guidance and demonstrated a mean motion of 0.3 mm transversely and < 3.75 mm cranio-caudally. Biro et al. [20] reported a reduction in liver motion from 20 to 5 mm following switching from IPPV to HFJV, and liver motion < 3 mm has been reported during HFJV-assisted hepatic radiotherapy using fiducial markers [22]. Diaphragmatic excursion is therefore minimal compared with gross motion using standard IPPV tidal volumes [18], and HFJV assistance appears to provide effective respiratory immobilisation.

HFJV assistance appeared to be safe, with no episodes of desaturation, hypercapnia, or barotrauma. One patient experienced bronchospasm requiring temporary conversion to IPPV; however, this was due to insufficient depth of anaesthesia, and HFJV was promptly recommenced following additional anaesthetic administration. There were no differences in technical success/outcomes compared with IPPV. Use of current-generation systems (which continuously monitor airway pressure with an alarm signal to avoid barotrauma) and intermittent CO_2_ monitoring with HFJV further increases procedural safety [14]. HFJV also proved practical and widely applicable, with simple/user-friendly ventilator installation and few contra-indications (COPD with forced expiratory volume in the first second < 1500 mL, severe obesity, and recent pneumothorax/thoracic surgery [32]). However, the need for specially trained anaesthesiology teams, and potentially longer anaesthetic times are aspects that should be taken into account [14, 21, 23, 24].

Study limitations include small sample size, including only few patients undergoing PAVM embolisation under general anaesthesia, and short follow-up, limiting generalisability and outcome comparison. Groups were non-randomised, but there were no significant differences in demographics, lesions, and embolisation devices. Procedural duration did not include anaesthetic time, and there was no evaluation of anaesthesiologist technical difficulty due to the retrospective protocol. Finally, diaphragmatic excursion was used as a surrogate for PAVM motion and measured using a gross method. Although non-comparable to standardised diaphragmatic measurements and without proven correlation with PAVM motion, this was sufficient to illustrate significant relative motion reduction with HFJV assistance compared with IPPV.

In conclusion, in patients undergoing PAVM embolisation under general anaesthesia, HFJV-assisted embolisation is a safe, practical technique enabling respiratory immobilisation and improved lesion targeting thus resulting in reduced patient radiation dose and procedural time. Larger prospective randomised studies are required to confirm the short- and long-term effects of this resurgent anaesthetic technique in vascular interventional procedures.

## Data Availability

The dataset supporting the conclusions of this article is included within the article.

## Abbreviations

2D: Two-dimensional
3D: Three-dimensional
CBCT: C-arm cone-beam CT
CECT: Contrast-enhanced computed tomography
CT: Computed tomography
ETCO_2_: End-tidal carbon dioxide
FiO_2_: Fraction of inspired oxygen
HFJV: High-frequency jet ventilation
HHT: Hereditary haemorrhagic telangiectasia
IPPV: Intermittent positive pressure ventilation
MVP: Microvascular plug
PAVM: Pulmonary arteriovenous malformation
SpO_2_: Peripheral oxygen saturation
